# Developing a routine lab test for absolute quantification of HER2 in FFPE breast cancer tissues using Quantitative Dot Blot (QDB) method

**DOI:** 10.1038/s41598-020-69471-4

**Published:** 2020-07-27

**Authors:** Guohua Yu, Wenfeng Zhang, Yunyun Zhang, Jiahong Lv, Shishou Wu, Xiaolong Sui, Jiandi Zhang, Fangrong Tang

**Affiliations:** 1 Lab of Molecular Pathology, Dept of Pathology, Affiliated Yuhuangding Hospital of Qingdao University, Yantai, Shandong People’s Republic of China; 2 Yantai Quanticision Diagnostics, Inc., a Division of Quanticision Diagnostics, Inc., of USA, Yantai, Shandong People’s Republic of China; 3 Center for Precision Medicine, Binzhou Medical University, Yantai, Shandong People’s Republic of China; 4 Quanticision Diagnostics, Inc., 400 Park Offices Dr., Research Triangle Park, NC 27709 USA

**Keywords:** Biological techniques, Biotechnology, Cancer, Molecular biology, Biochemistry, Immunochemistry, Proteins, Biomarkers, Diagnostic markers, Predictive markers, Prognostic markers, Oncology, Breast cancer, Tumour biomarkers

## Abstract

Developing immunoassay for absolute quantitation of protein biomarkers in Formalin Fixed Paraffin Embedded (FFPE) samples promises improved objectivity, consistency and accuracy in daily clinical practice. The feasibility of Quantitative Dot Blot (QDB) method for this purpose was explored in this study. We were able to measure HER2 protein levels using 0.5 µg/sample total protein lysate extracted from 2 × 5 µm FFPE slices absolutely and quantitatively using QDB method in 332 breast cancer FFPE samples. HER2 levels measured using two clinically validated antibodies for immunohistochemistry respectively were highly correlated (r = 0.963). We also achieved area under the curve (AUC) at 0.9998 ± 0.0002 (p < 0.0001, n = 224) with IHC analysis, and 0.9942 ± 0.0031 (p < 0.0001, n = 319) with combined results from IHC and *Fluorescence *in situ* hybridization* (FISH) analyses when analyzed with Receiver Operative Characteristics analysis (ROC) respectively. When the results were converted dichotomously with optimized cutoffs from ROC analyses, we achieved 99.5% concordance with IHC; and 96.9% with combined results from both IHC and FISH analyses. Therefore, we were able to demonstrate QDB method as the first immunoassay platform for absolute quantitation of protein biomarkers in FFPE samples to meet the need of daily clinical practice, especially for local laboratories or laboratories in developing countries.

## Introduction

Although Immunohistochemistry (IHC) is the prevailing method in protein biomarker assessment for solid tumors, the inherent problems, including the lack of objectivity and consistency, are also well recognized in the field^1–3^. While intensive efforts have been devoted to the standardization of this method^1, 4–7^, there are also ongoing efforts to develop alternative methods for absolute quantitation of biomarker protein levels objectively and consistently for routine clinical practice.

Considering that the majority of clinical samples are preserved as Formalin Fixed Paraffin Embedded (FFPE) block in pathological practice, one pre-requisite for any method suitable for routine clinical practice is that this method must be compatible with FFPE samples. In this regard, the Enzyme linked Immunosorbent Assay (ELISA) method is not a feasible option. The low binding capacity of ELISA plate (400–600 ng/cm^2^) limits the amount of bound antigen for further analysis due to heavy protein crosslinking in FFPE samples, and the concentrating effect of capture antibody in Sandwich ELISA is also nullified for the same reason.

Reverse Phase Protein Microarray (RPPA) analysis has been reported to measure the expression levels of several protein biomarkers in FFPE samples^8, 9^. However, its results are relative, not suitable for comparison among experiments. In addition, its complicated analytical processes and high costs limit its usage in routine clinical practice. For the same reason, although Selected Reaction Monitoring Mass Spectrometry (SRM-MS) has become the only method to measure several protein biomarkers successfully in FFPE samples absolutely and quantitatively^10–12^, it is not an ideal option in daily clinical practice, especially in the local clinical laboratories and clinical labs in developing countries.

Recently, Quantitative Dot Blot (QDB) method has been developed in our company as an improvement over the traditional antibody-based, dot blot method to allow high throughput, absolute quantitation of a specific protein at tissue level^13–16^. By introducing a nitrocellulose membrane-based 96-unit QDB plate in the assay, this method significantly increases the protein-binding capacity of individual unit (100 to 200 µg/cm^2^) to meet the analytical need of immunoblot analysis. The consistency, sensitivity and accuracy of the results are also increased by counting the luminescence signal directly in a microplate reader.

To develop a QDB-based assay is also very straightforward. It requires minimum time and effort for assay optimization, as the time-proven buffers used in Western blot analysis can be adopted directly in a QDB-based assay. The high binding capacity of nitrocellulose membrane also eliminates the need of concentrating antigen as in Sandwich ELISA. Admittedly, the antibody pair used in Sandwich ELISA may achieve higher specificity than a QDB-based assay, as only one detection antibody is used in a QDB-based assay. Nonetheless, a validated antibody used in other immunoblot processes, including IHC, flow cytometry, and Western blot analysis with single band at detection, can be used directly in a QDB-based high throughput assay**.** In a proof of concept (POC) study, we have measured HER2 levels quantitatively and absolutely in frozen breast cancer tissues with QDB method using clinically validated antibodies for IHC (IHC antibodies)^15^.

HER2 (HER2/Neu or ERBB2) is one of the most used protein biomarkers among breast cancer patients^7^. Overexpression of this protein has been found among 20–30% invasive breast patients^8^. Targeted therapies against HER2 protein, represented by Trastuzumab (Herceptin), have found success in treating patients testing HER2 positive (HER2+), but not with those testing negative (HER2−)^9^.

Currently, HER2 level is assessed mainly through IHC. Based on recommendations from American Society of Clinical Oncology/College of American Pathologists **(**ASCO/CAP), HER2 level is scored as 0, 1+ , 2+ and 3+ , with those scoring 0 and 1+ being defined as HER2−, and those 3+ as HER2+ . Samples scored as 2+ are defined as equivocal, requiring further differentiation by Fluorescence in situ hybridization (FISH) analysis^10, 11^. While IHC analyzes HER2 at protein level, FISH is used to analyze HER2 amplification at DNA level, and is considered the golden standard for assessing HER2 level in FFPE specimen^17^. However, this method is more complicated, requiring more processing time than IHC analysis.

In our POC study, when HER2 protein levels were converted into dichotomous variables (HER2+/HER2−) using Receiver Operative Characteristics analysis (ROC), we found QDB results were highly consistent with IHC analysis results. Furthermore, results from QDB analysis eliminate the equivocal cases, as measured HER2 levels were either above or below the proposed cutoff value in our study.

The simplicity, objectivity and consistency of QDB method with frozen samples demonstrate its potential use in daily clinical practice. This method also liberates the pathologist from judging the IHC results under the microscope. However, it remains unclear if the QDB method can be extended to FFPE samples to overcome the heavy crosslinking of proteins in these samples.

In this study, we measured HER2 levels in 332 FFPE samples with QDB method using EP3 and 4B5, the same two IHC antibodies used in our POC study. The consistency of results was demonstrated by comparing the results using two different IHC antibodies, EP3 and 4B5 antibodies, and by comparing with those from both IHC and FISH analyses using Receiving Operative Characteristics (ROC) analysis. The correlation between HER2 protein level and several clinicopathologic parameters was also evaluated statistically.

## Results

### Assay development and quantitative measurement of HER2 levels in 332 FFPE samples

**Figure 1:**
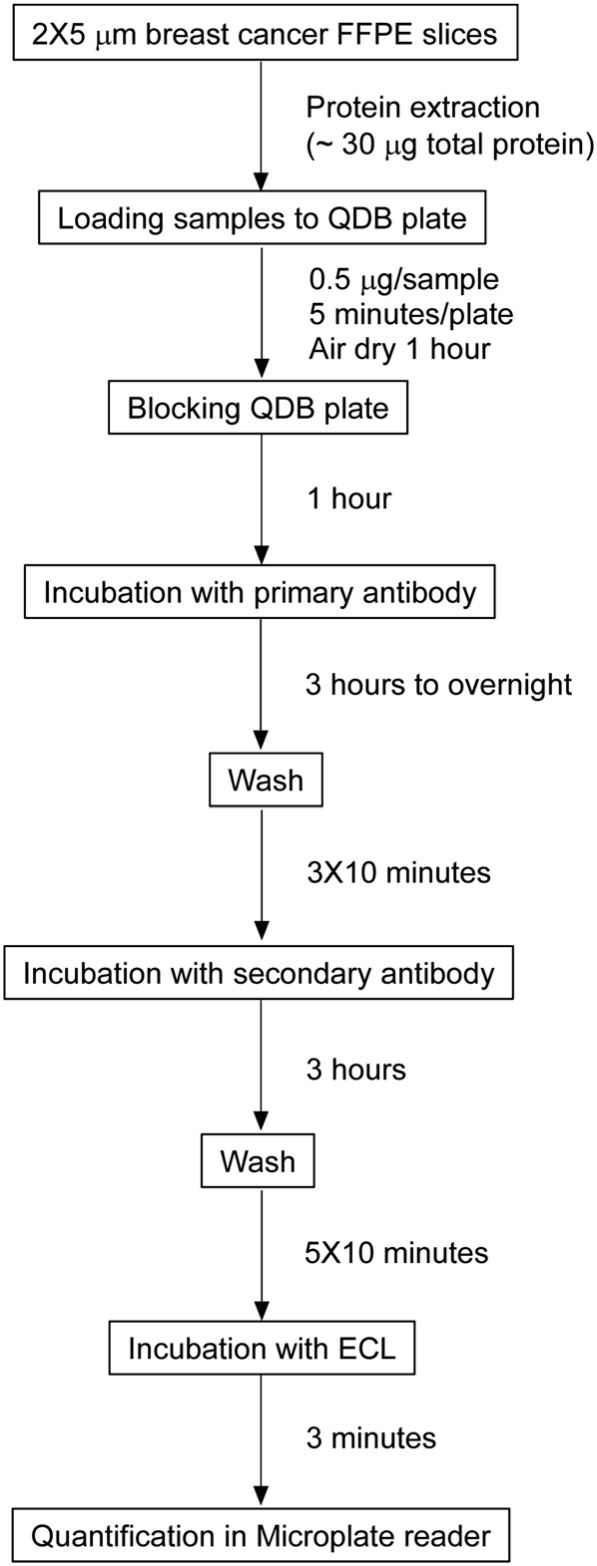
The flow chart of QDB process.

We first developed QDB-based immunoassay for HER2 measurement in FFPE samples (for flow chart, see Fig. 1). The linear ranges of the assay were explored using pooled lysates of HER2+ specimens and recombinant HER2 protein in serial dilutions in QDB measurement with both 4B5 and EP3 antibodies respectively (Supplementary Fig. 1).

**Figure 2:**
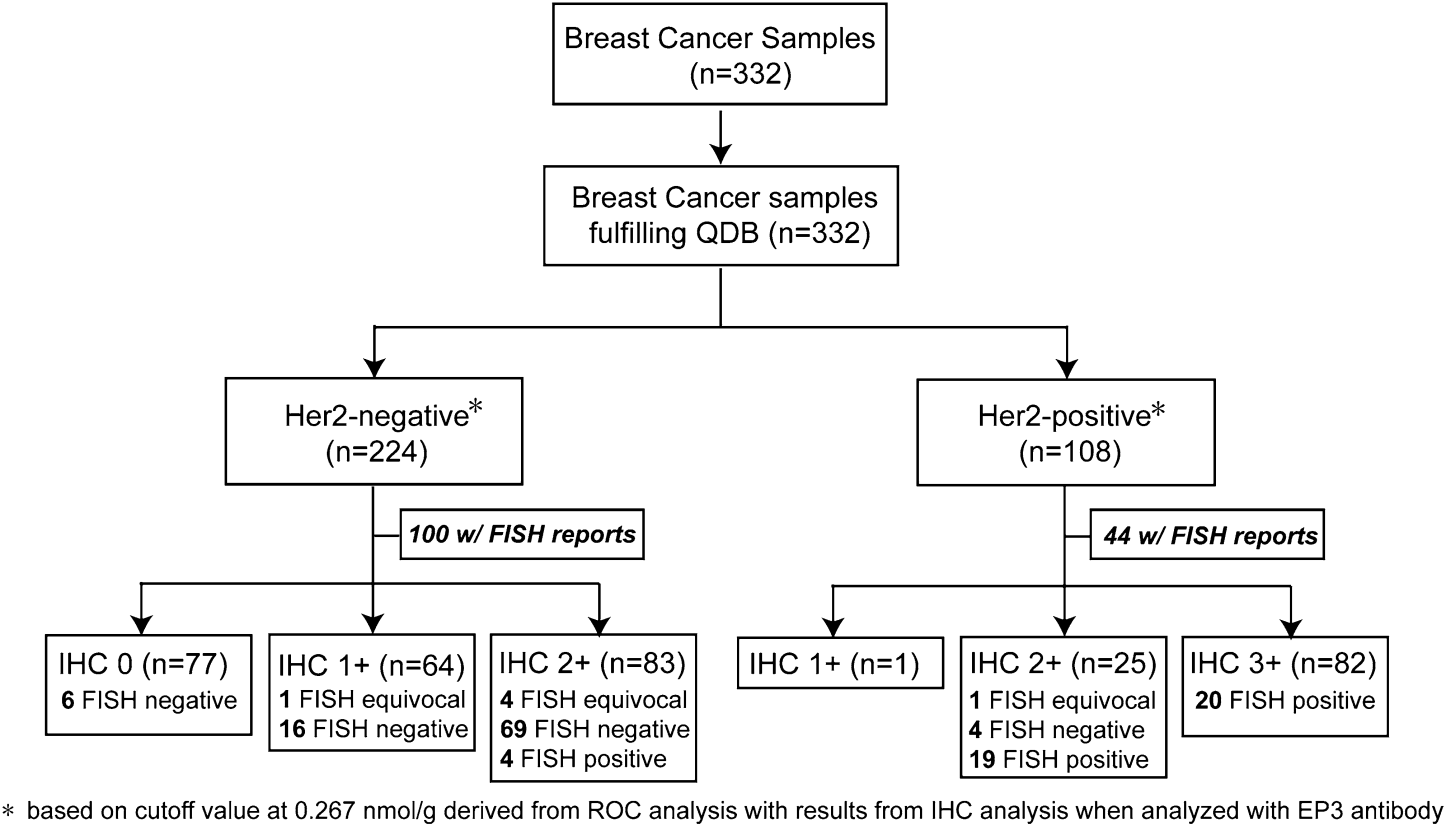
The flow diagram of participants.

**Figure 3:**
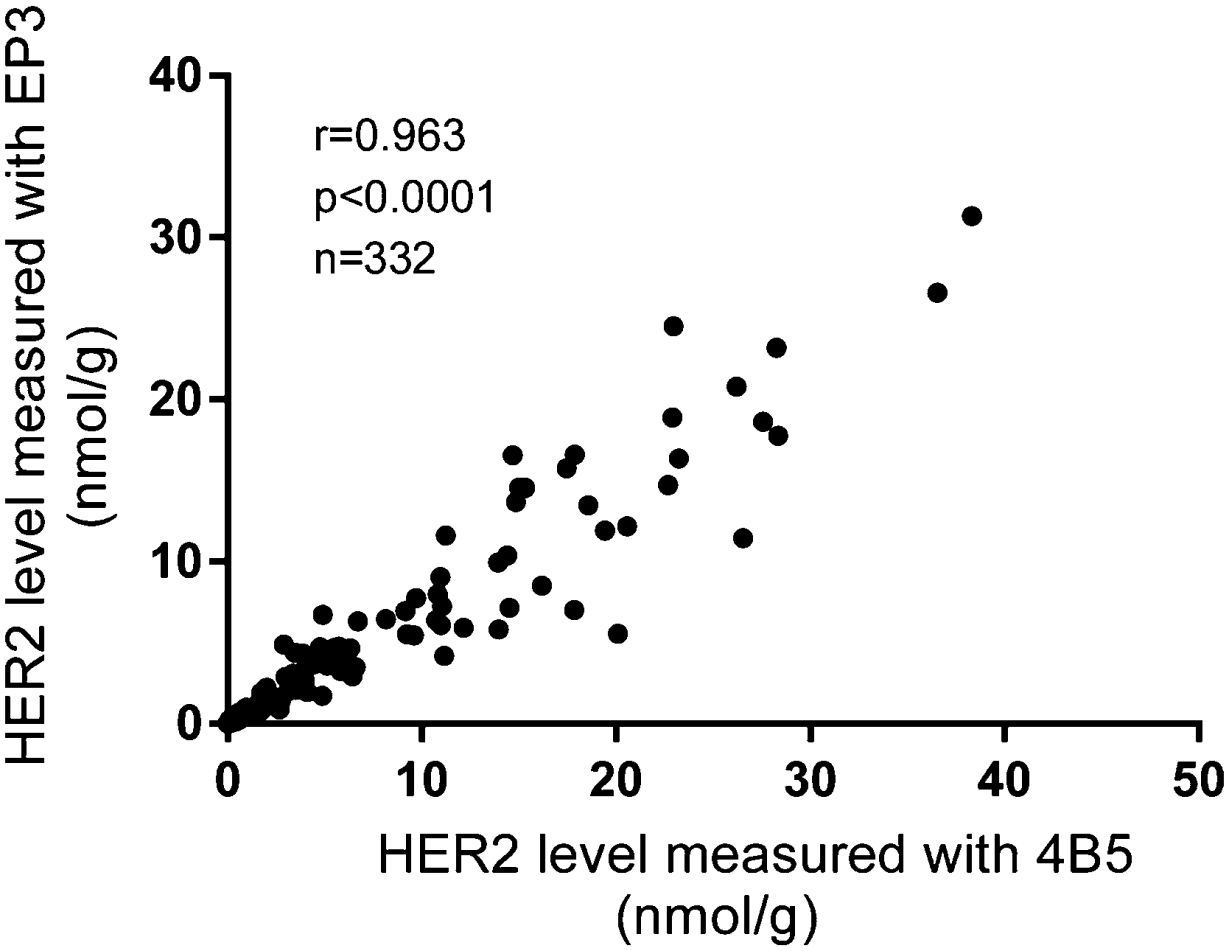
Correlation of HER2 levels measured with 4B5 and EP3 antibodies. A total of 332 breast cancer FFPE tissues in 2 × 5 µm slices were provided by a local hospital. MCF-7 and BT474 cell lysates were used as internal controls. FFPE tissue lysates (about 0.5 µg/unit) and cell lysates (about 0.3 µg/unit) were applied onto the QDB plates at 2 µl/unit in triplicate for the QDB measurements with clone EP3 and 4B5 respectively. A set of serially diluted HER2 recombinant protein were included in each plate to develop plate-specific standard curve. All results were averaged from three independent experiments, with each sample in triplicate. The correlation of HER2 levels measured with 4B5 and EP3 was analyzed with Pearson’s correlation coefficient analysis using Graphpad software, r = 0.963, p < 0.0001.

The HER2 protein levels were measured using total tissue lysates extracted from 332 FFPE samples with both 4B5 and EP3 antibodies within their defined linear range respectively. Samples were provided as 2 × 5 µm FFPE slices sequentially and non-selectively by local hospital with clinicopathological characteristics listed in Table 1. The flow diagram is shown in Fig. 2. HER2 levels measured with 4B5 and EP3 antibodies were highly correlated, with r = 0.963, p < 0.0001, n = 332 when evaluated with Pearson’s correlation coefficient analysis (Fig. 3). For simplicity, we limited our analysis in this study to HER2 levels measured with EP3 antibody.

**Table 1:** The clinicopathological characteristics of the patients.

Variable	No. of patients	Average ± SEM	Percentage (%)
**Age (years)**			
Total	332	53.3 ± 0.6	
< 50	122		36.7
≥ 50	209		63.0
Unknown	1		0.3
**Histological grade**			
I	36		10.8
II	145		43.7
III	119		35.9
Unknown	32		9.6
**Tumor size (cm)**			
Total	332	2.3 ± 0.6	
≤ 2	173		52.1
2–5	151		45.5
> 5	5		1.5
Unknown	3		0.9
**Histological type**			
Ductal	298		89.8
Lobular	9		2.7
Other	23		6.9
Unknown	2		0.6
**Nodal status**			
Negative	220		66.3
Positive	112		33.7
**HER2 (IHC)**			
0	77		23.2
1+	65		19.6
2+	108		32.5
3+	82		24.7
**HER2 (FISH)**			
Negative	95		28.6
Equivocal	6		1.8
Positive	43		13.0
Unknown	188		56.6

**Figure 4:**
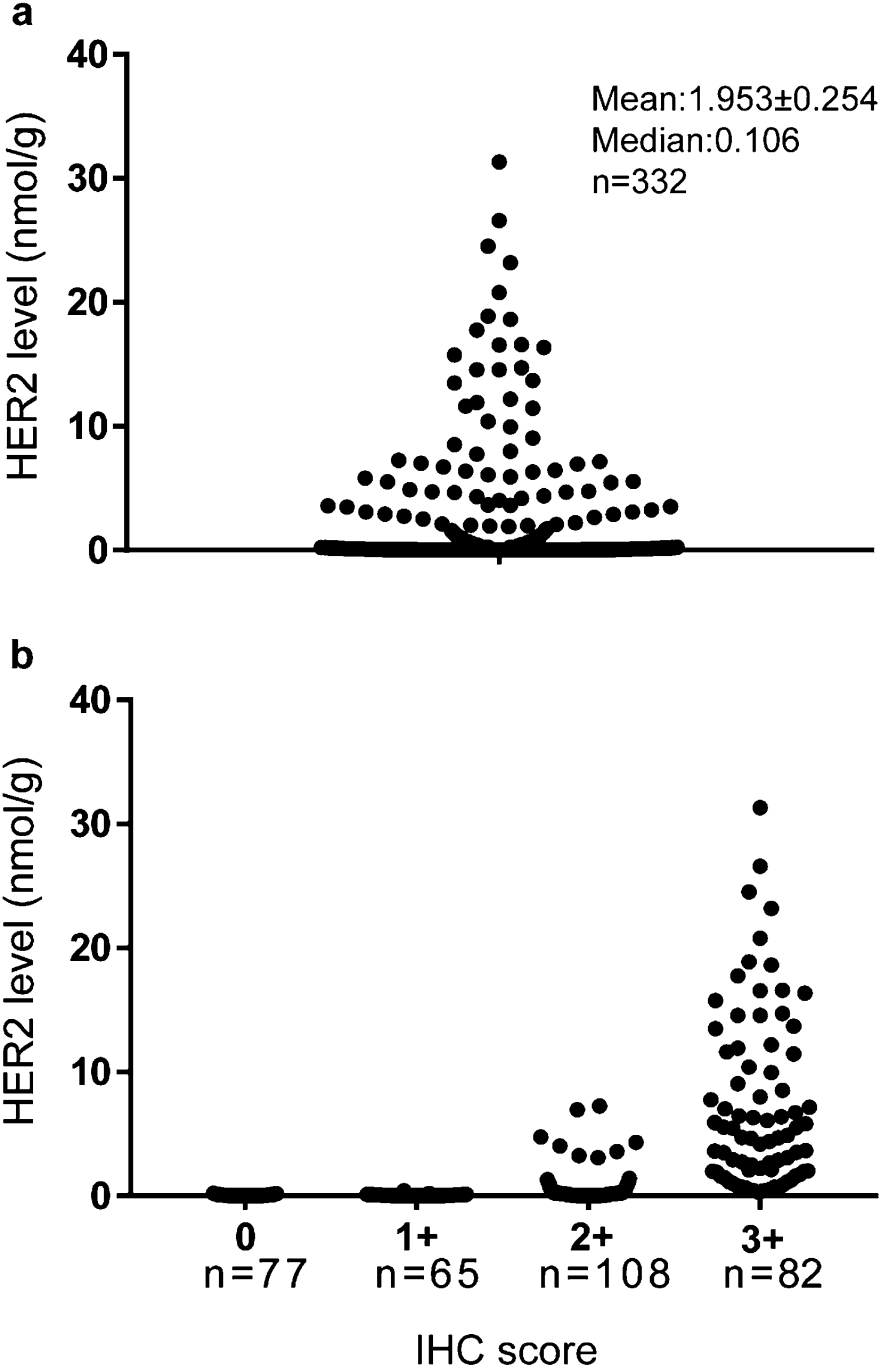
Distribution of all 332 samples. HER2 levels in all 332 breast cancer FFPE sample lysates were measured with the QDB method using EP3 antibody. The lysates were diluted to about 0.25 µg/µl, and then 2 µl lysate was used for each sample. (**a**) the distribution of HER2 levels among 332 samples. HER2 levels ranged from 0 (chemiluminescence readings less than two fold over the background) to 31.310 nmol/g. (**b**) All samples were grouped by their IHC scores provided by local hospital. The distributions of HER2 levels in each IHC group were recorded as following: 0, 0–0.205 nmol/g, mean = 0.045 ± 0.006 nmol/g, n = 77; 1 +, 0 –0.410 nmol/g, mean = 0.049 ± 0.008 nmol/g, n = 65; 2+ , 0–7.250 nmol/g, mean = 0.537 ± 0.122 nmol/g, n = 108; and 3+ , 0.329–31.310 nmol/g, mean = 7.120 ± 0.773 nmol/g, n = 82. The intra- and inter-CV were 8.98% and 9.89% respectively.

The distribution of HER2 levels among these samples was shown in Fig. 4a. The absolute HER2 level was distributed from non-detectable to as much as 31.31 nmol/g. The samples were grouped into 0, 1+, 2+ and 3+ groups based on IHC scores with average at 0.045 ± 0.006 (n = 77), 0.049 ± 0.008 (n = 65), 0.537 ± 0.122 (n = 108), and 7.120 ± 0.773 (n = 82) nmol/g respectively (Fig. 4b). The differences between each individual group were statistically significant with one exception when analyzed using two-tailed Student’s t-test (p < 0.005). There was no statistical difference between group 0 and group 1+ .

### Validation of QDB method

The only method for absolute quantification of HER2 levels was SRM-MS, which was still in developmental stage^10, 12, 18^. Consequently, we had to rely on the results from both IHC and FISH analyses to validate our results indirectly, as the results from the QDB method were quantitatively measured, while those from FISH and IHC analyses were category classified. Therefore, Receiver Operative Characteristics (ROC) analysis was used to evaluate QDB method with IHC and FISH analyses respectively.

**Figure 5:**
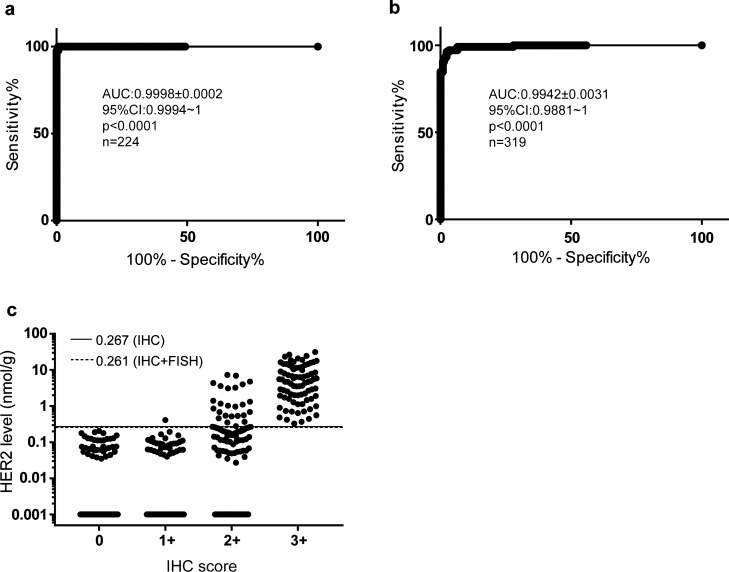
Evaluation of QDB results with those of IHC and FISH analyses using ROC analysis. Samples were separated into negative (HER2−) and positive (HER2+) groups based on the recommendations from ASCO/CAP. In (**a**), samples were grouped based on their IHC scores, with 142 samples in the negative group (IHC 0 and 1+), and 82 samples in the positive group (IHC 3+). Absolute HER2 levels from these samples were used for ROC analysis with Graphpad Prism7.0 software. The ROC curve of QDB results was obtained with area under the Curve (AUC) at 0.9998 ± 0.0002; 95% CI 0.9994–1; p < 0.0001. In (**b**), samples were grouped based on both IHC and FISH results, with 214 samples as negative (HER2−) group and 105 samples as positive (HER2+) group. 6 equivocal cases and 7 cases with missing FISH results were excluded in the analysis. Absolute HER2 levels from these samples were used for ROC analysis with Graphpad Prism7.0 software. The area under the curve (AUC) was at 0.9942 ± 0.0031, with 95% CI at 0.9881–1; p < 0.0001. (**c**) The samples were grouped by IHC scores, and the suggested cutoff values from ROC analyses in (**a**) at 0.267 nmol/g (solid line), and in (**b**) at 0.261 nmol/g (dashed line) were shown to demonstrate the effectiveness of these cutoff values to separate samples from HER2+ to HER2− groups. HER2 levels were plotted in log scale to better demonstrate the distribution of QDB results among these samples. For those samples with undetectable HER2 level, a value of 0.001 nmol/g was arbitrarily entered to avoid omitting any sample in the log scale graph.

The samples were grouped into HER2− (0 and 1+) and HER2+ (3+) groups based on IHC analysis for ROC analysis. Those 2+ samples were excluded in the analysis, as these samples are defined as equivocal, requiring further analysis with FISH. We determined area under the ROC curve (AUC) at 0.9998 ± 0.0002, 95% CI at 0.9994 to 1, with p < 0.0001 (n = 224) (Fig. 5a). We also identified the optimized cutoff at 0.267 nmol/g to achieve 100% sensitivity (95% CI 95.6% to 100%) and 99.3% specificity (95% CI 96.14% to 99.98%) with IHC results.

Among 144 samples with determined FISH results, 101 samples were with IHC score of 2+ to be excluded from ROC analysis with IHC results. Therefore we considered FISH results as an independent validation of the optimized cutoff determined from ROC analysis. Using 0.267 nmol/g as this cutoff, we were able to achieve 88.6% concordance rate with FISH analysis (κ = 0.732, n = 144 with Cohen’s kappa analysis), with 16 samples (11.4%) identified in disagreement with provided FISH results. To rule out potential misdiagnosis, these samples were submitted to a third party for independent FISH analysis (Supplementary Table 1). We were able to re-categorize 6 samples, with 2 samples as equivocal case. Obviously, 50% (8/16) of original FISH results were questioned by both QDB method and third party analysis.

Next, clinical testing results from both IHC and FISH analyses were combined, and used to evaluate the overall performance of QDB method using ROC analysis independently. As shown in Fig. 5b, we were able to achieve area under the ROC curve (AUC) at 0.9942 ± 0.0031, with 95% CI at 0.9881 to 1, p < 0.0001, n = 319. At optimized cutoff of 0.261 nmol/g, we achieved 97.14% sensitivity (95% CI 91.88% to 99.41%) and 96.74% specificity (95% CI 93.38% to 98.67%). The overall concordance rate was 96.9% (Fig. 5c). It should be mentioned that we used revised FISH results in the analysis, and those equivocal cases from FISH analysis were excluded from analysis.

The correlation between HER2 copy numbers from FISH analysis, reflected by the ratio of HER2 number over chromosome 17 number (HER2/CEP17), with quantitated HER2 protein level was analyzed in Supplementary Fig. 2. We found a strong correlation between DNA amplification level and HER2 protein level, with r = 0.75 with Pearson’s correlation coefficient analysis (n = 122).

### Exploration of the correlation between clinicopathologic factors and quantitated HER2 protein levels

The quantitated HER2 levels in FFPE samples allows us to investigate its correlation with other clinicopathologic factors including age, histological grade by Nottingham grading system, tumor size and metastasis status. HER2 levels were found to be associated significantly with histological grade based on Nottingham grading system using Spearman’s rank correlation analysis (ρ = 0.195, p = 0.001), a conclusion consistent with previous studies based on IHC analysis^19, 20^. In the same study, we found that age was negatively associated with HER2 with statistical significance based on IHC analysis (ρ = − 0.117, p < 0.05), but not based on the absolutely quantitated HER2 levels (ρ = − 0.084, p = 0.127) (Supplementary Table 2).

**Figure 6:**
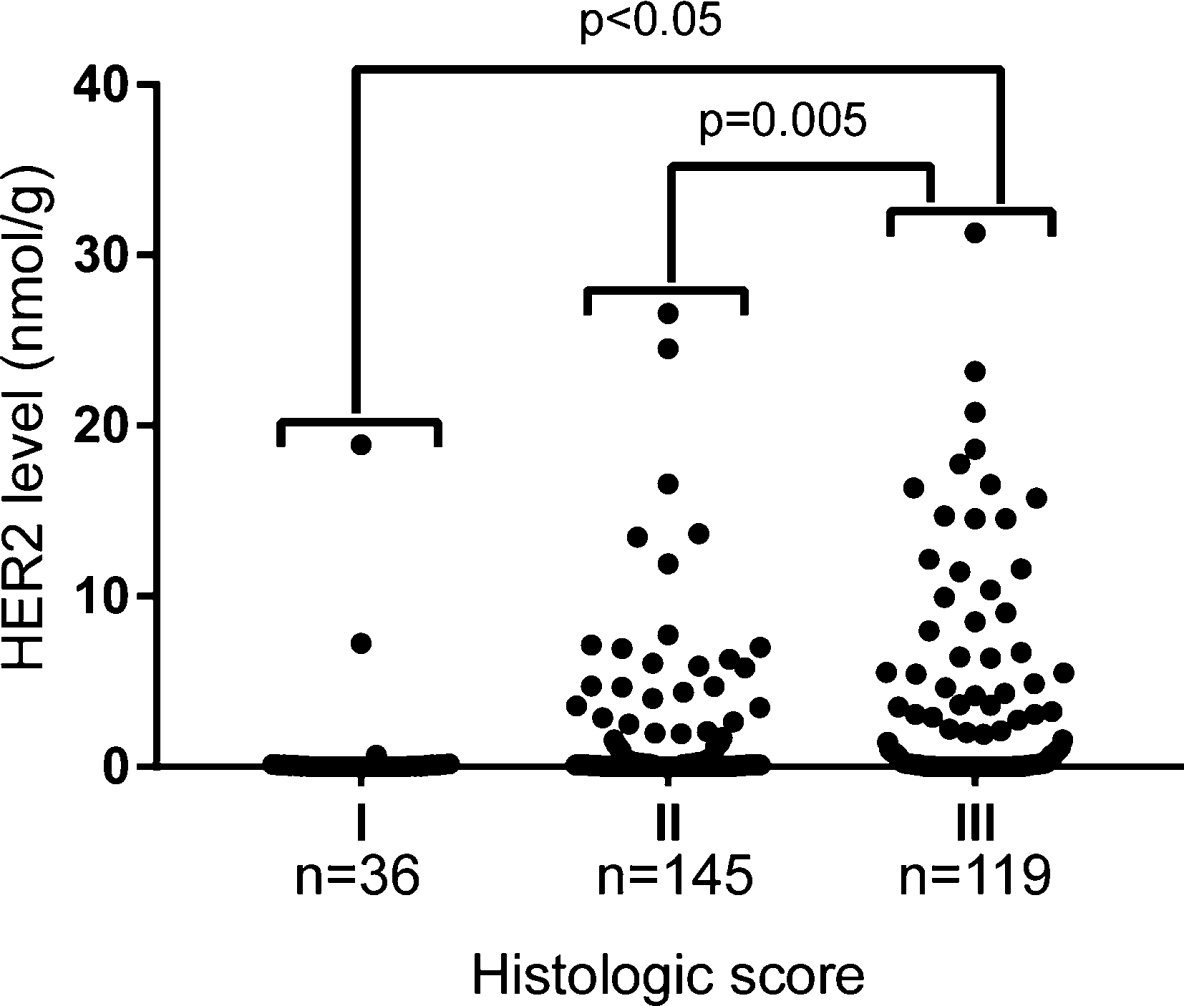
Assessing HER2 levels by histologic grade. The FFPE specimens (300 out of 332) were grouped according to their Nottingham histologic scores into Grades I, II, and III. The HER2 levels of each grade were used for column statistics analysis with Graphpad Prism7.0 software. The mean ± SD of the HER2 levels were 0.791 ± 0.555 nmol/g for Grade I (n = 36), 1.554 ± 0.330 nmol/g for Grade II (n = 145), and 3.271 ± 0.535 nmol/g for Grade III (n = 119). The statistical difference was assessed with two-tailed Student’s t-test, with p < 0.05 between Grades I and III, and p = 0.005 between Grades II and III. There was no statistical difference between Grade I and Grade II samples.

HER2 distribution by histological grade was further analyzed in Fig. 6. We observed the average of these samples by Grades at 0.791 ± 0.555, 1.554 ± 0.330, 3.271 ± 0.535 nmol/g for Grade I, Grade II, and Grade III respectively. There were statistical significance in the differences between Grade I vs Grade III (p < 0.05) and between Grade II vs Grade III (p = 0.005) using two-tailed Student’s t-test. We also calculated the percentage of HER2+ in each grade with 8.3% for Grade I, 29.7% for Grade II and 47.1% for Grade III. Thus, the possibility of HER2+ for Grade III patient was 5.7 fold over that of Grade I patient.

## Discussion

In this study, we measured HER2 protein levels absolutely and quantitatively in 332 FFPE samples using the QDB method with two clinically validated antibodies for IHC analysis. The results from this high throughput method are objective and consistent. When converted into dichotomous variables, results from QDB method showed high concordance with both FISH and IHC analyses. It is worth to mention that there were no equivocal cases in QDB measurements, as the results were either above or below the proposed cutoff, thus significantly reducing the ambiguity associated with the current prevailing methods of FISH and IHC analyses.

To the best of our knowledge, this is the first time protein biomarker was measured absolutely and quantitatively in FFPE samples using an immunoassay. The assay requires minimum amount of total protein lysates extracted from FFPE slices (0.5 µg per sample for HER2). At this rate, lysates prepared from 2 × 5 µm slices from a typical FFPE block were sufficient to analyze over 10 protein biomarkers. The method also required minimum training to be practiced in a routine clinical laboratory settings. The inherent validation steps in the analytical process, including using cell lysates with pre-determined HER2 content as quality control, also significantly reduced the inter-laboratory variations.

All these features support QDB method as a suitable platform to develop immunoassays for daily clinical practice, especially for local laboratories, and clinical laboratories in developing countries. In addition, this transition may be accelerated significantly by QDB’s ability to use clinical validated antibodies (IHC antibodies) directly in the assay development process. We believe that the adoption of this method in daily clinical practice will be a major step in the standardization of the assessment of protein biomarkers in FFPE samples, considering the issues plaguing the current prevailing methods of IHC analysis.

It should be emphasized that QDB is not a replacement of IHC. Rather, it complements IHC in accurately assessing the protein levels of tissue biomarkers in daily clinical practice. Clearly, IHC remains irreplaceable when addressing morphological issues relating to the cellular localization and the intratumoral heterogeneity of tissue biomarkers. Nonetheless, the semi-quantitative nature of IHC analysis makes it a mission impossible to quantify the protein levels of tissue biomarkers accurately and consistently.

In this regard, intratumoral heterogeneity of protein biomarkers bring more challenges to the quantitation of protein biomarkers in IHC analysis. QDB method, on the other hand, is able to reduce its interference to a minimum to provide an overall assessment of protein biomarker levels in the tissue. Obviously, both methods are needed to provide an accurate and comprehensive assessment of tumor heterogeneity in the tumor tissue.

The QDB method and IHC analysis are both based on antigen–antibody interaction. Ideally, results from QDB analysis should match very well with those from IHC analysis when IHC analysis is performed properly as dichotomous variables. However, this method is fundamentally different from FISH analysis, as one analyzed at protein level (QDB), while the other one analyzed at DNA level (FISH). It is well recognized that the FISH analysis has the inherent drawback of being unable to reflect faithfully changes at protein level^21^. In fact, in the prescribing information for OGIVRI, a biosimilar for Herceptin from Mylan GmbH, out of total 1,272 patients testing HER2+ based on IHC analysis (3+), 51 was found HER2− in FISH analysis, with another 51 unknown, thus making the concordance between FISH and IHC at 95.8% excluding the unknown cases^22^. In our study, the concordance would be 94.1% if the results from the third party was incorporated in the analysis. Considering one major usage of HER2 assessment is for antibody-based targeted therapy to block HER2 action at protein level**,** we believe QDB results are more clinically relevant than those from FISH analysis.

In this study and several other studies, HER2 levels showed wide distribution even among HER2 positive samples^10, 23–25^. The highest level we measured in QDB analysis was more than 100 fold over the proposed cutoff value with both antibodies. Furthermore, studies based on SRM-MS method showed that both gastric and breast cancer patients with higher level of HER2 responded better to Herceptin treatment^2, 10^. These results strongly demonstrate the necessity of quantifying HER2 levels quantitatively among cancer patients^5^.

In conclusion, we developed the first immunoassay for absolute quantitation of HER2 levels in FFPE samples. The success of this study supports QDB method as a reliable platform for absolute quantitation of tissue biomarkers in FFPE specimen for routine clinical use. Considering the large number of FFPE samples available worldwide, this study may open door to a new area in clinical diagnosis, where the clinical usage of tissue biomarkers will be fully explored through database-supported mathematical analysis at population level.

## Methods

### Human subjects and human cell lines

A total of 332 Formalin Fixed Paraffin Embedded (FFPE) specimens in 2 × 5 µm slices treated for breast cancer between Jan. 2015 and Aug. 2017 were provided sequentially and non-selectively by Yuhuangding Hospital at Yantai, P. R, China. All the samples were obtained in accordance with the Declaration of Helsinki, and approved by ethics committee of Yuhuangding hospital ([2017]76 to Guohua Yu) with an informed consent waiver due to the use of archival tissues with retrospective, anonymized clinical data.

MCF-7 and BT474 cell lines were purchased from the Cell Bank of Chinese Academy of Sciences (Shanghai, China), and maintained according to the provider’s instruction.

### General reagents

All of the chemicals were purchased from Sinopharm Chemicals (Beijing, P. R. China). Recombinant human HER2/ErbB2 protein was purchased from Sino Biological Inc. (Beijing, China). Ventana anti-HER2/neu (4B5) rabbit monoclonal primary antibody was purchased from Roche Diagnostics GmbH. Rabbit anti-HER2 antibody (clone EP3) was purchased from ZSGB-BIO (Beijing, China). HRP labeled Donkey Anti-Rabbit IgG secondary antibody was purchased from Jackson Immunoresearch lab (West Grove, PA, USA). BCA protein quantification kit was purchased from Thermo Fisher Scientific Inc. (Calsband, CA, USA). QDB plate was provided by Quanticision Diagnostics, Inc. (RTP, USA).

### Preparation of FFPE tissue and cell lysates

Two FFPE tissue slices at 5 µm each (2 × 5 µm) were put into 1.5 ml Eppendorf tubes, and deparaffinized before they were solubilized using lysis buffer (50 mM HEPES, 137 mM NaCl, 5 mM EDTA, 1 mM MgCl_2_, 10 mM Na_2_P_2_O_7_, 1% TritonX-100, 10% glycerol) with protease inhibitors (2 µg/ml Leupeptin, 2 µg/ml Aprotinin, 1 µg/ml Pepstatin, 2 mM PMSF, 2 mM NaF). MCF-7 and BT474 cells were also lysed in the same lysis buffer. The supernatants were collected after centrifugation and the total amount of protein was determined using BCA protein assay kit.

### QDB analysis

The QDB process was shown in Fig. 1. It was also described elsewhere with minor modifications^13, 14^. In brief, the final concentration of the FFPE tissue lysates was adjusted to 0.25 µg/µl, and 2 µl/unit was used for QDB analysis in triplicate. The loaded QDB plate was dried in the air for 1 h, or at 37 °C for 30 min before it was blocked in blocking buffer (4% non-fat milk in TBST) for an hour. The plate was inserted into a 96-well microplate filled with 100 µl/well primary antibody (for clone EP3, 1:1,500 in blocking buffer; for clone 4B5, 1:10 in PBS), and incubated overnight at 4 °C. Afterward, the plate was rinsed twice with TBST and washed 3 × 10 min before it was incubated with a donkey anti-rabbit secondary antibody for 3 h at RT. The plate was rinsed twice with TBST, washed 5 × 10 min and then was inserted into a white 96-well plate pre-filled with 100 µl/well ECL working solution for 3 min. The chemiluminescence signals of the combined plate were quantified using Tecan Infinite 200pro Microplate reader with the option “plate with cover”.

BT474 and MCF-7 cell lysates with pre-documented HER2 level were included in all the experiments to ensure the consistency of the results. The results were accepted only when measured HER2 levels were within 10% of pre-determined levels. Samples with chemiluminescence reading less than twofold over blank were defined as non-detectable, entering 0 for data analysis. For those samples with the chemiluminescence reading less than that of 30 pg HER2 recombinant protein, the narrow range (0–125 pg) linear regression formula was used to calculate low HER2 level.

### FISH analysis

A total of 16 samples were submitted to ZSGB-Bio, Inc (https://www.zsbio.com) at Beijing, China for FISH analysis. The detailed reports are available upon request.

### Statistical analysis

All the data were presented as Mean ± SEM. The difference between individual groups was calculated using two-tailed Student’s t test. A p value < 0.05 was considered statistically significant. The correlation analysis was performed using either Pearson’s correlation coefficient analysis or Spearman’s rank correlation analysis as indicated in the figure. The performances of QDB method against IHC or FISH were evaluated using Receiver Operative Characteristics (ROC) analysis. All statistics were performed using GraphPad Prism software version 7.0 (GraphPad Software Inc., USA).

### Ethics declarations

All the study, including samples collection and study protocol prepared and followed, was in accordance with the Declaration of Helsinki, and approved by ethics committee of Yuhuangding hospital ([2017]76 to Guohua Yu) with informed consent waiver for anonymized archival tissues with retrospective clinical data.

## Supplementary information

Supplementary Information 1.

## Data Availability

The data will be available upon request by writing to Jiandi.zhang@outlook.com.
