# Supplementary material for: Developing a routine lab test for absolute quantification of HER2 in FFPE breast cancer tissues using Quantitative Dot Blot (QDB) method
Source: Sci Rep. 2020 Jul 27;10:12502. doi: 10.1038/s41598-020-69471-4 (PMC7385113; doi:10.1038/s41598-020-69471-4)
Supplement: Supplementary file 1 — Supplementary Information 1. [file 41598_2020_69471_MOESM1_ESM.docx]

**Developing a routine lab test for absolute quantification of HER2 in FFPE breast cancer tissues using Quantitative Dot Blot (QDB) method**

*Short title:* *biomarker quantitation in FFPE tissue with QDB*

Guohua Yu^1#^, Wenfeng Zhang^2^, Yunyun Zhang^2^, Jiahong Lv^2^, Shishou Wu^1^, Xiaolong Sui^1^, Jiandi Zhang^2,3*^, and Fangrong Tang^2#^.

**Supplementary Fig. 1：Defining the linear range of QDB measurements with two anti-HER2 antibodies, EP3 and 4B5.**

**
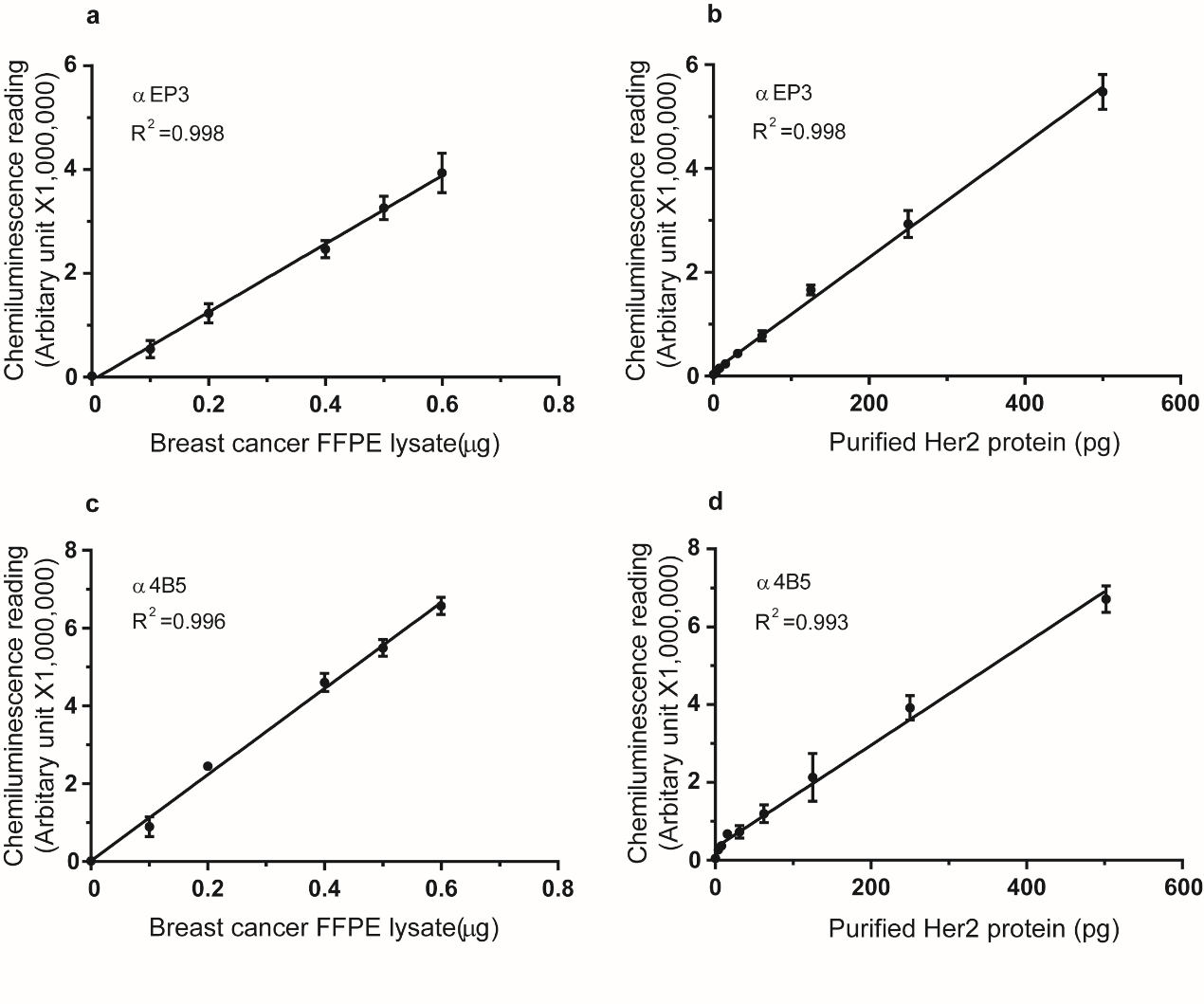
**

(**a**, **c**) Defining the linear range of QDB method for analysis of breast cancer FFPE tissue lysates. Human breast cancer FFPE tissue blocks in two 5 µm slices (2X5 µm) were obtained from a local hospital, and the tissue lysates were prepared as described in Methods. Breast cancer FFPE tissue lysates prepared from 4 samples with an IHC score of 3+ were mixed in equal amounts based on the BCA assay. The pooled lysate was serially diluted as indicated in the figure with 0.5 µg/µl IgG-free BSA solution to ensure equal loading of the samples. The lysates were then applied onto the QDB plate at 1 µg/unit in triplicate for QDB analysis using two anti-HER2 antibodies, EP3 and 4B5 respectively. (**b**, **d**) Defining the linear range of QDB method for analysis of purified HER2 recombinant protein. The HER2 recombinant protein was serially diluted with 0.5 µg/µl IgG-free BSA solution. The diluted solution was then used for QDB analysis at 1 µg/unit in triplicate for measurement with EP3 and 4B5 antibodies respectively.

**Supplementary Fig. 2：Correlation between HER2 gene copy numbers (HER2/CEP17) and their protein levels.**

**
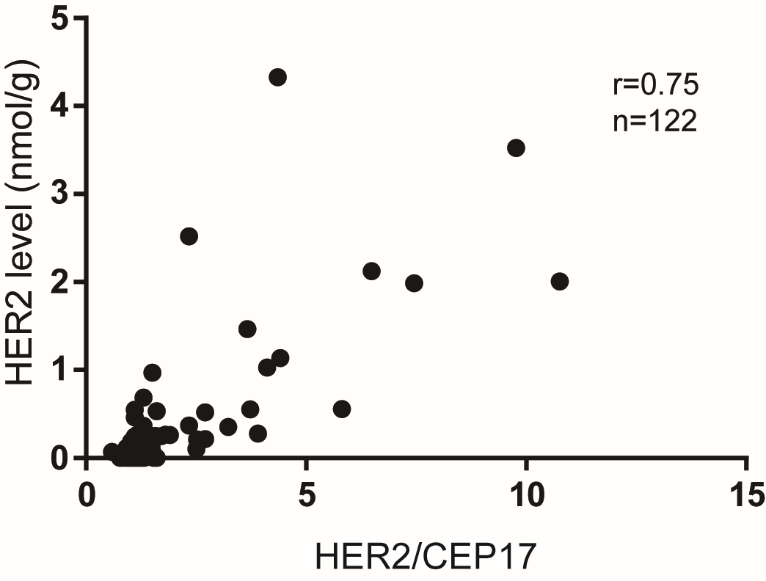
**

The correlation was assessed with Pearson correlation coefficient analysis using HER2 protein levels from QDB analysis and HER2/CEP17 ratio from FISH analysis (r= 0.75, p<0.0001). The statistical analysis was performed using Graphpad 7.0.

**Supplementary Table. 1:** **Third party verification samples inconsistent with locally provided FISH results**

| **Sample NO.** | **FISH**  *(local hospital)* | **QDB**  *(nmol/g)* | **FISH**  *(Third party)* |
| --- | --- | --- | --- |
| 290  79  119  141  298  149  293  294  159 | negative | **0.547**  **0.459**  **0.686**  **0.365**  **0.681**  **0.531**  **0.520**  **0.849**  **0.967** | Equivocal  Negative  Negative  Negative  Negative  **Positive**  **Positive**  **Positive**  **Positive** |
| 22  96  286  64  288  218  301 | **positive** | 0.068  0  0.050  0.102  0.212  0.213  0.262 | Equivocal  Negative  Negative  **Positive**  **Positive**  **Positive**  **Positive** |

The HER2 levels measured with QDB method were used to separate samples into HER2+ and HER2- using suggested cutoff value at 0.267 nmol/g. There were 16 samples identified with disagreed QDB and FISH results. These samples were sent to a third party to rule out potential misdiagnosis, and the results were shown at the right column. All the positive results, either from FISH or QDB analyses, were in bold. The concordance rate would be improved to 94.2% (κ=0.865 using Cohen’s Kappa analysis) when the results from third party were incorporated in the analysis.

**Supplementary Table. 2: Assessment of relationships between clinicopathologic features and HER2 levels by QDB and IHC analysis respectively.**

| **Variable** | **Age** | **Histological Grade** | **Tumor size** | **Nodal status** |
| --- | --- | --- | --- | --- |
| **QDB** | -0.084 | 0.195*** | 0.039 | -0.041 |
| **IHC** | -0.117* | 0.204*** | 0.087 | -0.019 |

HER2 levels, assessed either by QDB method as absolute and continuous variables, or IHC analysis as relative and discrete variables, were used to explore the putative association with clinicopathological features using Spearman’s rank correlation analysis using Graphpad 7.0. The statistically significant associations were indicated in the figure. The histological grades based on Nottingham grading system were found to be associated with HER2 levels assessed either by IHC (ρ=0.195, p<0.001) or QDB measurement (ρ=0.204, p<0.0005) with statistical significance. * p<0.05; *** p<0.001.
